# Transcription factor and miRNA co-regulatory network reveals shared and specific regulators in the development of B cell and T cell

**DOI:** 10.1038/srep15215

**Published:** 2015-10-21

**Authors:** Ying Lin, Qiong Zhang, Hong-Mei Zhang, Wei Liu, Chun-Jie Liu, Qiubai Li, An-Yuan Guo

**Affiliations:** 1 Hubei Bioinformatics & Molecular Imaging Key Laboratory, Huazhong University of Science and Technology, Wuhan, 430074, China; 2 Department of Bioinformatics and Systems Biology, Huazhong University of Science and Technology, Wuhan, 430074, China; 3 Key Laboratory of Molecular Biophysics of the Ministry of Education, Huazhong University of Science and Technology, Wuhan, 430074, China; 4 College of Life Science and Technology, Huazhong University of Science and Technology, Wuhan, 430074, China; 5 Institute of Hematology, Union Hospital, Tongji Medical College, Huazhong University of Science and Technology, Wuhan 430022, China

## Abstract

The maturation process of lymphocyte was related to many blood diseases, such as lymphoma and lymphoid leukemia. Many TFs and miRNAs were separately studied in the development of B and T cells. In this study, we aim to discover the TF and miRNA co-regulation and identify key regulators in the B and T cells maturation. We obtained the candidate genes, miRNAs and TFs for each stage of their maturation, then constructed the TF-miRNA-gene feed-forward loops (FFLs) for each stage by our previous methods. Statistical test for FFLs indicated their enrichment and significance. TF-miRNA co-regulatory networks for each stage were constructed by combining their FFLs. Hub analysis revealed the key regulators in each stage, for example, MYC, STAT5A, PAX5 and miR-17 ~ 92 in the transition of pro-B cells into pre-B cells. We also identified a few common regulators and modules in two stages of B cell maturation (e.g. miR-146a/NFKB1/BCL11A) and two stages of T cell maturation (e.g. miR-20/CCND2/SORL1), as well as some shared regulators in the early stages of both B and T cell development. Our network will help to increase understanding of mature process of B and T cell, as well as the related blood diseases.

Hematopoiesis is the formation of all kinds of blood cells and an ideal model to study multi-lineage differentiation in humans^1^,^2^. Lymphocytes, as the cornerstone of the adaptive immune system, are derived from common lymphoid progenitors (CLP) and primarily composed of T cells and B cells^3^. The development of T cells within the thymus is a complex process including stages double negative (DN), double positive (DP) and CD4 or CD8 single positive (SP) T-cells^4^. The B cell maturation should undergo sequential developmental stages including pro-B cells, pre-B cells, immature B cells and mature B cells^5^,^6^. Studying their development and differentiation will be helpful to better understand the hematopoiesis and pathogenesis of blood disorders.

There are various genes involved in the mature processes of B cell and T cell. Earlier, two groups respectively studied several stages of gene expression profiles in the development of normal human B cell and T cell by using DNA microarray, which revealed the gene expression characteristics in the development of lymphocytes^5^,^7^. As the main regulators of gene expression, microRNAs (miRNAs) and transcription factors (TFs) play key roles in the development of lymphocytes and a few studies were published. Studies have shown that miRNAs play important roles in the cell development of immune system, such as miR-34a inhibits the transition of pro-B cells into pre-B cells by inhibiting FOXP1^8^. MiR-181a has shown to be a decisive factor in tuning the TCR signaling threshold by targeting multiple phosphatases, i.e. PTPN22, DUSP5, DUSP6 and SHP-2^9^. TFs are paramount regulators of gene expression in living organisms^10^, and also involved in lymphoid cell development. For example, FOXP3 is required for differentiation into CD4+ Treg cells by regulating miR-155 expression^11^. E2A, EBF1 and PAX5 have been considered as key TFs in B-lymphopoiesis^12^,^13^,^14^.

TF and miRNA may mutually regulate each other to form feed-back loops (FBLs) or feed-forward loops (FFLs) in which a TF regulates a miRNA or a miRNA represses a TF, and both of them co-regulate a joint target^15^. The FFL is a significant network motif in genome and many of them were identified and validated. The MYC-miR-26a-EZH2 FFL has been recognized in aggressive B-cell lymphomas and was associated with lymphoma aggressive progression^16^. Another study also evaluated a critical role of NF-κB/STAT5/miR-155 network in FLT3-ITD-driven acute myeloid leukemia^17^. The FOXP3-miR-7/miR-155-SATB1 was important to prevent the transformation of the healthy breast epithelium to a cancerous phenotype^18^. Our previous studies have highlighted the importance of miRNA and TF co-regulatory networks in T-cell acute lymphoblastic leukemia, schizophrenia and myocardial infarction^19^,^20^,^21^. Considering that established studies about the development of lymphocytes were mainly on the regulation of TFs or miRNA separately, we attempted to combine the TF and miRNA to study their co-regulatory network in the mature processes of B cell and T cell.

In this study, by curating the lymphopoiesis related candidate miRNAs and TFs from literatures, screening and identification of differentially expressed genes from GEO DataSet, we constructed a comprehensive miRNA-TF-gene (M-T-G) regulatory network. We compared the similarities and differences between B cell and T cell development, and identified key miRNAs, TFs and regulatory modules in their maturation. It may deepen our understanding of the differentiation process of the lymphoid cells.

## Results

### Candidate miRNAs, TFs and genes for every stage of the B or T cell differentiation process

**Figure 1:**
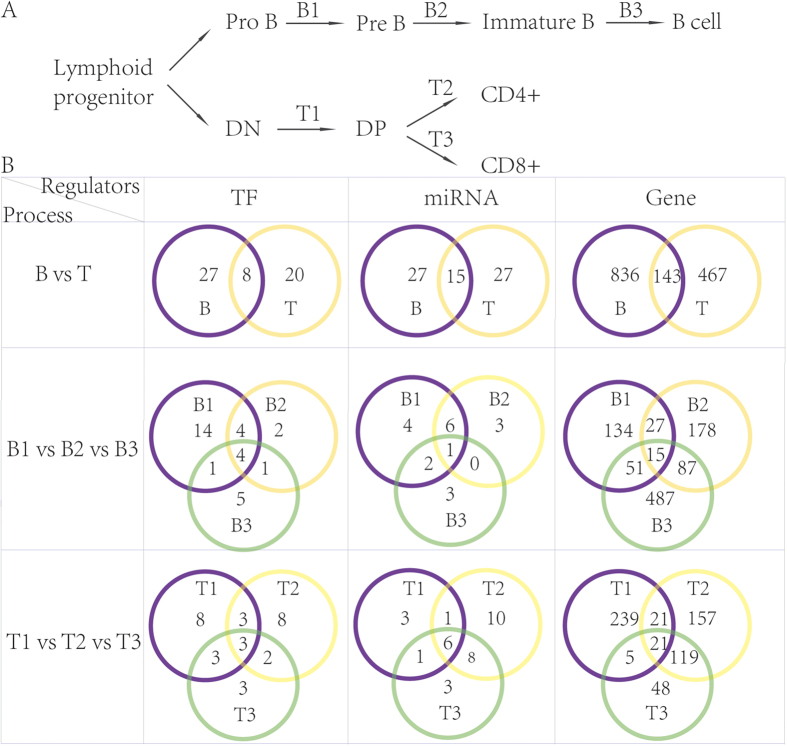
Candidate miRNA, TF and gene in each process of B and T cells maturation. (**A**) The process of (**B**) cells and T cells differentiation. (**B**) Venn diagram of miRNAs and TFs and genes in each process.

According to literatures, we defined the three main processes (stages) of B cell and T cell maturation as B1 (ProB-PreB), B2 (PreB-Immature B), B3 (Immature B-B) and T1 (DN-DP), T2 (DP-CD4+), T3 (DP-CD8+) (Fig. 1A). We collected verified TFs and miRNAs that play important roles in different processes, as well as the differentially expressed genes (Additional file 1). We found that there were many common regulators in the mature processes of B cell and T cell. Also there were some miRNAs and TFs involved in all three processes of B (or T) lineage. They may be the key factors to lymphocytopoiesis. On the other hand, there were some other specific regulators for every process (Fig. 1B).

We carried out functional enrichment analysis for differentially expressed genes, and found many significant enriched pathways (Supplementary Table S1). Taking the B lineage as an example, B cell receptor signaling pathway was a typical pathway in the B1 stage. B2 stage mainly involved in the Cell cycle, and B3 stage mainly involved in apoptosis, etc. We also found that some TFs were exactly the differentially expressed genes of the corresponding process, e.g. MYC, STAT5A and MYB (Supplementary Dataset1). All these indicated that the data we collected were reliable.

### TF and miRNA feed-forward loops in the lymphoid cell development

FFLs can be divided into three types according to the master regulators: TF-FFL, miRNA-FFL and composite FFL^15^. In Table 1, we summarized the FFLs in each stage, and found the TF-FFL was the dominant type in the development of B cells. It is notably that many composite-FFLs appear in the T2 and T3 stages, implying the processes more complex. We tested the significance of FFLs in each stage by random simulation test (see Method). As shown in Table 1, all P-values were less than 0.05, indicating that the FFLs observed in the set of candidate TFs, miRNAs and differentially expressed genes were significantly enriched against genome background. It also proved that the collected miRNAs and TFs are relevant to the lymphopoiesis.

### The miRNA-TF co-regulatory network for every stage of the B or T cell differentiation process

**Figure 2:**
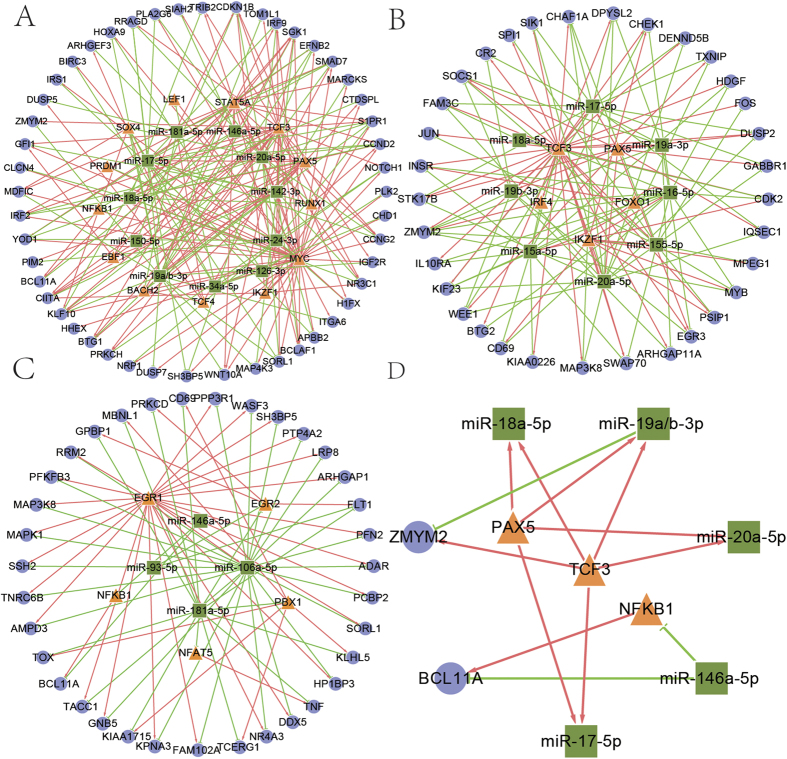
Network and core regulatory relationships in B cell maturation. (**A**) Network of B1 stage. (**B**) Network of B2 stage. (**C**) Network of B3 stage. (**D**) Common modules in B lineage. Orange triangles: TFs. Green rectangle: miRNAs. Blue circular: genes. The edge colors represent different relationships: green for regulation of miRNAs to genes or TFs, red for the regulation of TFs to genes or miRNAs.

Based on the identified FFLs, we constructed the miRNA-TF co-regulatory network for each stage of the development process of B and T cells. The network for B1 stage (called B1 network as following, and the same for other networks) has the most nodes and edges, because more miRNAs involved in this stage (Fig. 2A, Table 1). The B2 network shared many miRNAs and TFs with B1 network (Fig. 2B), which suggesting the development of B cells is a stepwise process. B3 network consists of three isolated sub-networks, in which EGR1 and miR-106a–5p co-regulated many genes to form the largest sub-networks (Fig. 2C). Some nodes and edges involved in more than one processes, we defined the regulatory module among them as common modules (Fig. 2D). Such as the regulatory relationships between PAX5/TCF3 and miR-17/18a/19a/19b/20 appeared in the stages of B1 and B2, and a lot of literatures showed their regulation in the early development of B cells^14^,^22^,^23^. The sub networks of miR-146a/NFKB1/BCL11A appear in the B1 and B3 stages, which indicated this FFL may be very important to the development of B lineage.

**Figure 3:**
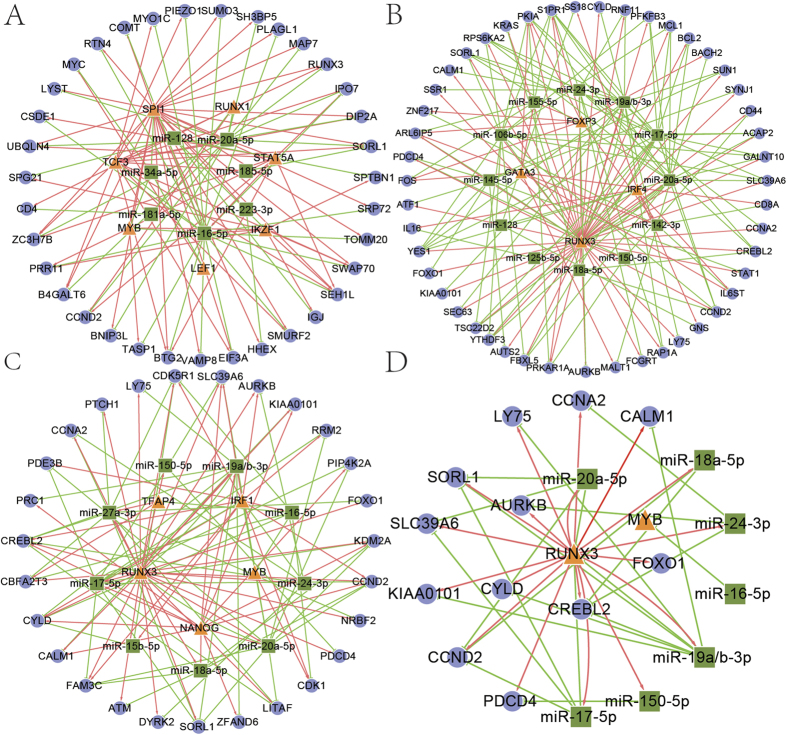
Network and core regulatory relationships in T cell maturation. (**A**) Network of T1. (**B**) Network of T2. (**C**) Network of T3. (**D**) Common modules in T lineage. The means of different nodes and edges are the same as Fig. 2.

The miRNA and TF regulatory networks for T1, T2 and T3 stages (called T1, T2, T3 network respectively) of the T cell maturation were shown in Fig. 3A–C and Table 1. As shown in Fig. 3, TFs in RUNX, IRF, MYB and GATA families are usually the key roles in the network of T cell maturation. Due to more composite-FFLs in this process, T3 network has less nodes, but more edges (Fig. 3C, Table 1). As shown in Fig. 3D, T2 and T3 networks shared many common nodes, such as miR-17 ~ 92 cluster and RUNX3, which indicated that they may be important to determine the CD4+/CD8+ lineage. A previous study reported that RUNX factors were involved in CD4 silencing and also directly affected positive transcriptional control of CD8^24^. In addition, miR-20/CCND2/SORL1 were a common FFL in the 3 stages and miR-16 regulates MYB appeared in the T1 and T3 network, suggesting their important roles in the maturation of T cells, and many studies proved these^25^,^26^.

### Hubs of the networks are key regulators in the B and T cell maturation

Hubs are highly connected in a network, thus they play critical roles in maintaining the structure and function of the network. As shown in Table 2, in the B1 network, PAX5/STAT5A/MYC and miR-17/19/20/24 were the hub regulators. And MYC regulating miR-17 ~ 92 cluster, as the most obvious hub miRNA-TF pair, regulated majority genes in the network. It is consistent with a previous study that MYC upregulates miR-17 ~ 92 expression and contributes to B-cell development^27^. As the only hub TF in the B2 network, TCF3 formed miRNA-FFLs with hubs miR-15/16 and target genes, and it also generated TF-FFLs with members of miRNA-17 ~ 92 cluster, miR-155 and target genes. Literature survey results also showed that TCF3 was crucial to the mature of B cell (Supplementary Table S2). B3 network has less hubs, and hub regulators EGR1 and miR-106a–5p co-regulated many genes of the T cell receptor signaling pathway, such as MAPK1, MAP3K8 and PPP3R1.

In the T1 process, SPI1 was the hub TF regulating most genes in the network (Table 2, Fig. 3A). As reported, SPI1 was necessary to full proliferation, restricting access to some non-T fates, and controlling the timing of T-cell developmental progression^28^. STAT5A, TCF3 and miR-20a/16 were also the hub regulators of this network. In the T2 network, RUNX3, as key regulator in T cell maturation, was a hub TF and formed many composite-FFLs with miRNAs in miR-17 ~ 92 cluster in both T2 and T3 processes. In the process of T3, NANOG and RUNX3 were two hub TFs formed many TF-FFLs with other miRNAs and genes. Members in miR-17 ~ 92 cluster were often hub miRNAs in the network and miR-20a was a hub miRNA shared by three stages, which indicated it was very important to the maturation of T cells.

### Key regulators in the early of B and T cell maturation

**Figure 4:**
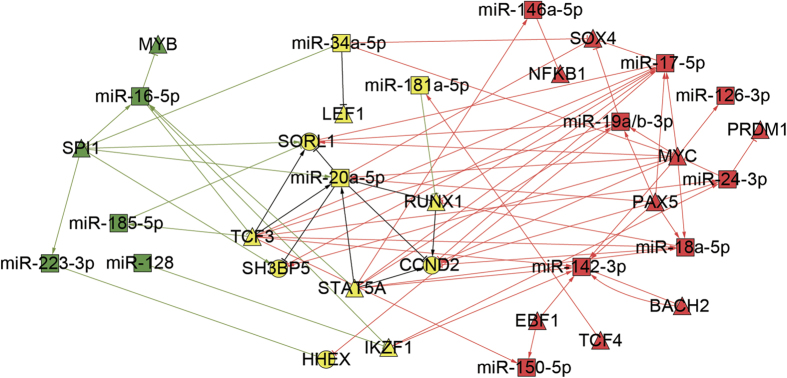
Shared and specific regulators in T1 and B1 network. Yellow nodes in the middle are the shared regulators and genes in T1 and B1 network; Green nodes represent regulators specifically in T1 network; Red nodes are regulators specifically in B1 network.

In order to better understand the relevance and similarity between the development of T cell and B cell, we analyzed the important regulatory elements in the B1 and T1 stages. As shown in Fig. 4, miR-17 ~ 92 cluster, miR-150 and PAX5, EBF1 etc. play leading roles in B1, while miR-223/16/128, MYB and SPI1 are the key regulators in the early stage of the development of T cells. There were also many shared miRNAs and TFs in the B1 and T1 stage, indicated that the developments of T and B cells were not two independent processes. For example, miR-181a/20a/34a, RUNX1, STAT5 and TCF3 were involved in both B1 and T1 stage (Fig. 4). They regulated different target genes in the two processes and also regulated a few shared target genes, such as CCND2, SH3BP5 and HHEX. Multiple studies suggested that abnormal expression of CCND2 involved in the pathogenesis of leukemia^29^,^30^.

## Discussion

Lymphocyte development is a complex biological process, we investigated the co-regulation of miRNAs and TFs involved in this process. By integrating various data, we constructed TF-miRNA co-regulated FFLs and network in every stage of lymphocyte development, and then identified the key regulators and FFLs in every stage (Table 1). In order to guarantee the accuracy of the data, we took the various data sources to check each other. The enriched pathway of differentially expressed genes (Supplementary Table S1) showed that lymphocyte self-renewal ability was becoming weaker and the differentiation ability was getting stronger along with the lymphocyte development. Permutation test suggested the constructed FFLs was significantly different from random results (Table 1). All these results illustrated the accuracy of TFs and miRNAs we collected from literature, so the FFLs based on them was credible.

Recent studies have shown that regulations by miRNAs and TFs are tightly coupled in FFLs^15^,^19^,^20^,^31^, which may work as the ‘core’ of the whole gene regulatory network. The key nodes obtained by further analysis of network topology may be important regulators in the corresponding stage. According to the number of B/T cells-related papers for every candidate TF and miRNA (Supplementary Dataset1), we found that hubs in every network were studied by many papers, and most hubs were the top 10 most studied TFs/miRNAs (Supplementary Table S2, S3). This proved the importance of our identified hubs in another way. For example, our analysis showed PAX5, STAT5 and TCF3 were hub nodes and important TFs in the B1 stage (Fig. 2A), and a lot of papers reported that they were closely related to the early development of B cells^32^,^33^. Hubs miR-16/15b in T3 network has been shown to be highly expressed in cells CD8+^34^.

At the same time, we also found that many regulatory modules in our network are consistent with previous reports. For example, MYC upregulates miR-17 ~ 92 expression and contributes to tumorigenesis and B-cell development^27^. Our results also revealed that MYC regulated miR-17 ~ 92 in the stage of B1. Besides, miR-155 and miR-15 regulated MYB in the stage of B2 in our results Fig. 3B and previous studies^35^,^36^. In the stage of T2, FOXP3 regulated miR-155/142, which is in accordance with the result that FOXP3 induced miR-155, and inhibited miR-142^37^,^38^.

We analyzed the similarities and differences in different stages of B and T cell differentiation processes. Some regulators were involved in more than one processes of B or T cell maturation, which may be crucial for their developments. Except for shared nodes in both T2 and T3 networks, there were also many process-specific regulators in the stages of T2 and T3. For example, miR-16/15b, which only contributed to the development of CD8+ cells, had been proven highly expressed in CD8+ cells^34^. NANOG, as a key TF of T3 process, regulated miR-17 ~ 92 cluster and their target genes to promote the maturation of CD8+ cells^39^. GATA3, which regulated genes related to TCR signaling pathway (e.g. CD8a), promoting the differentiation of DP into CD4+ but not CD8+^40^. They might be important regulators in determining CD4+/CD8+ lineage (T2/T3).

Our co-regulatory network will help to identify key regulators in the process of lymphocyte development and the regulatory modules among them. This study also had some limits, such as we only studied 3-nodes FFLs, but not 4-nodes FFLs^41^ and our current results were limited on the collected TF, miRNA and gene data from literature.

This study provided more details about molecular regulation mechanism in the process of lymphocyte development, and identified key regulators in every stage, which provided a new idea to understand the process of lymphocyte development, as well as diagnosis and treatment for blood disorders and cancers.

## Methods

### Candidate miRNAs, TFs and genes collection

To collect miRNAs and TFs involved in the mature processes of B cell and T cell, we conducted an extensive literature search. Firstly, we orderly searched PUBMED using the following keywords “miRNA AND hematopoiesis”, “(lymphopoiesis[Title/Abstract]) AND miRNA[Title/Abstract]”, “(B cell[Title/Abstract]) AND miRNA[Title/Abstract]”, and “(T cell[Title/Abstract]) AND miRNA[Title/Abstract]”. Then we manually read the retrieved publications and divided miRNAs into its corresponding process respectively. Finally, we removed the redundancies and obtained 6 miRNA lists for the 6 stages of B and T cell development. By the same way, we replaced the search keyword of “miRNA” to “transcription factor” and obtained candidate TFs for 6 stages. We also counted the numbers of B cell or T cell related papers for each candidate TF and miRNA.

Candidate genes for the B and T cells maturation were obtained from two gene expression profiles in the NCBI GEO database (GSE14714 and GSE22601), which were expression profiles of the corresponding stages of B cell and T cell development, respectively^5^,^7^. We screened differentially expressed genes of adjacent processes using the criteria of fold change >2 and false discovery rate (FDR) <0.01. These genes were mapped to the Entrez gene symbols and eventually we obtained 6 gene lists. Thus we obtained the candidate genes for each stage of B cell and T cell development.

The pathways overrepresented in candidate genes were analyzed by DAVID v6.7^42^. A p-value<0.01 was adopted as cutoff for enriched pathways in KEGG, and we only considered the top 6 as the representative pathways.

### Identification of miRNA and TF targets

TF targets were classified into two classes; (i) predicted targets and (ii) experimentally verified targets. For the predicted TF targets, we obtained predicted TFBS data from the UCSC genome browser (hg18 genome assembly) and set a Z score of 2.33 as a stringent cutoff for high value TFBSs (z-score ≥ 2.33, which is equivalent to a significance value of p < 0.01)^43^. Additionally, we also incorporated TF targets from ChIP-Seq and ChIP-chip data, which were curated from the ENCODE project^44^. Experimentally verified TF targets were extracted from TRANSFAC database (release 2013.4).

We merged experimentally verified and predicted miRNA targets as described in our previous review paper^15^. The experimentally verified targets were extracted from miR2Disease (Release Date: March 2011), miRTarBase (2013, version 4), miRecords (April 27, 2013) and TarBase (TarBase_V6, Jan 2012)^45^. These databases also contain many miRNA target relations based on the negative expression of miRNA and its targets from high-throughput experiments. For these miRNA targets based on negative expression, we also required the miRNA targets were predicted by both softwares TargetScan and miRanda to reduce the false positive rate.

### Feed-forward loops (FFLs) and statistics tests

The FFL is a network motif in which a TF regulates a miRNA or a miRNA targets a TF, and both of them co-regulate a gene^15^. According to the description of the above method section, we obtained candidate TFs, miRNAs and genes for each stage of B and T cell development, as well as the miRNA and TF targets. Thus, we can identify those TF-miRNA, TF-gene, miRNA-gene, miRNA-TF regulatory relations for each stage, and utilized in-house scripts to construct the miRNA-TF-gene FFLs for each stage. We using random permutation to evaluate if the FFLs observed in the set of candidate TFs, miRNAs and genes were significantly enriched against the genome background. For the permutation of TF, we fixed miRNAs and genes, and permutated TFs by randomly selecting TFs from all human TF set with the same number of our candidate TFs. Then, calculation of number of FFLs in the permutation network was performed. We repeated this 10000 times, and set the p-value as the proportion of the random permutation results that are less than the number of original FFLs. Next, we used the same method to permutate candidate miRNAs.

### Network and hub analysis

Based on the FFLs for each stage of B and T cell development, we constructed 6 miRNA-TF co-regulatory networks for these different processes. Then we also focused on several sub-networks, including B or T lineage common modules, which combined by the regulators involved in multiple processes of B or T cell maturation, respectively. All networks were visualized using Cytoscape (version 2.8)^46^. To determine hub TFs and miRNAs in networks, we counted the degree of each TF and miRNA node (sum of the in and out edges) in a network and calculated the proportion of out edges for that TF or miRNA. We defined the hub TFs and miRNAs as two requirements; (i) proportion of out edges is larger than average and (ii) the max number of hubs is 5. Similarly, the hub TF-miRNA pairs also satisfy two requirements: (i) The number of common target genes of the TF and miRNA is larger than average and (ii) The max number of hubs is 5.

## Additional Information

**How to cite this article**: Lin, Y. *et al.* Transcription factor and miRNA co-regulatory network reveals shared and specific regulators in the development of B cell and T cell. *Sci. Rep.* **5**, 15215; doi: 10.1038/srep15215 (2015).

## Supplementary Material

Supplementary file

Supplementary Dataset 1

## Figures and Tables

**Table 1:** Summary of the Characteristics of miRNA and TF co-regulatory network.

		Number of FFLs^a^	Statistical P value^b^		Network characteristics^c^			
Processes			TF	MiRNA	Edges	MiRNA	TF	Gene
B lineage	B1	172/19/1	0.0001	0	225	11	13	48
	B2	69/21/2	0.0068	0	128	7	5	33
	B3	24/12/0	0.0227	0.0185	77	4	5	35
T lineage	T1	43/17/0	0.0032	0.0001	109	7	7	35
	T2	28/26/56	0.0401	0	166	12	4	47
	T3	48/14/24	0.0135	0	116	9	5	27

^a^are the numbers of FFL for 3 types (TF-FFL/miRNA-FFL/composite-FFL).

^b^means the P-value of random permutation.

^c^means the number of edges or nodes.

**Table 2:** The hub components of each stage in the network in the B/T cell maturation process.

Hub	Pro B->pre B(B1)	Pre B->immature B(B2)	Immature B->B(B3)
TF	PAX5, STAT5A, MYC	TCF3	EGR1
miRNA	miR-19b–3p, miR-17–5p, miR-24–3p, miR-20a–5p, miR-19a–3p	miR-20a–5p, miR-17–5p, miR-16–5p, miR-15a–5p	miR-106a–5p, miR-181a–5p
TF-miR pair	MYC-miR-19–3pMYC- miR-20a–5pMYC- miR-17–5pSTAT5A- miR-20a–5pTCF3- miR-20a–5p	miR-15a–5p-TCF3miR-16–5p-TCF3TCF3- miR-155–5pTCF3- miR-20a–5p TCF3-miR-17–5p	EGR1- miR-106a–5pmiR-181a-5p-PBX1
**Hub**	**DN->DP(T1)**	**DP->CD4+(T2)**	**DP->CD8+(T3)**
TF	STAT5A, TCF3, SPI1	RUNX3	NANOG, IRF1, RUNX3
miRNA	miR-20a–5p,miR-16–5p	miR-19–3p,miR-20a–5p,miR-145–5p,miR-106b–5p,miR-17–5p	miR-27a–3p,miR-19–3p,miR-17–5p,miR-20a–5p
TF-miR pair	miR-16–5p-TCF3TCF3-miR-20aSTAT5A-miR-16–5pIKZF1- miR-16–5pSTAT5A-miR-20a–5P	*miR-20a–5p-RUNX3 miR-17–5p-RUNX3* miR-106b–5p-RUNX3 *miR-19–3p-RUNX3* miR-145–5p-RUNX3	RUNX3- miR-27a-3p*miR-17–5p-RUNX3miR-20a–5p-RUNX3miR-19–3p-RUNX3*NANOG- miR-19–3p

The hub TF-miRNA pair marked by italic represents mutual regulation between TF and miRNA.
