# Supplementary material for: Transcription factor and miRNA co-regulatory network reveals shared and specific regulators in the development of B cell and T cell
Source: Sci Rep. 2015 Oct 21;5:15215. doi: 10.1038/srep15215 (PMC4613730; doi:10.1038/srep15215)
Supplement: Supplementary file [file srep15215-s1.doc]

**Supplementary information**

**Transcription factor and miRNA regulatory network in the mature processes of B cell and T cell**

Ying Lin^1a^, Qiong Zhang^1a^, Hong-Mei Zhang^1^, Wei Liu^1^, Chun-Jie Liu^1^, Qiubai Li^2*^ and An-Yuan Guo^1*^

1 Hubei Bioinformatics & Molecular Imaging Key Laboratory, Department of Bioinformatics and Systems Biology, Key Laboratory of Molecular Biophysics of the Ministry of Education, College of Life Science and Technology, Huazhong University of Science and Technology, Wuhan, 430074, China

2 Institute of Hematology, Union Hospital, Tongji Medical College, Huazhong University of Science and Technology, Wuhan 430022, China

**Supplementary Tables**

**Supplementary Table S1: Canonical pathway enrichment of genes in the mature processes of B cell and T cell.**

| **Process** | **Pathway** | **P-value** |
| --- | --- | --- |
| Pro B->pre B(B1) | Primary immunodeficiency | 6.48E-04 |
| Pro B->pre B(B1) | Small cell lung cancer | 0.001521999 |
| Pro B->pre B(B1) | B cell receptor signaling pathway | 0.00407191 |
| Pro B->pre B(B1) | Jak-STAT signaling pathway | 0.004172886 |
| Pro B->pre B(B1) | Prion diseases | 0.005311191 |
| Pro B->pre B(B1) | Hematopoietic cell lineage | 0.00793928 |
| Pre B->immature B(B2) | Cell cycle | 4.03E-10 |
| Pre B->immature B(B2) | DNA replication | 2.57E-07 |
| Pre B->immature B(B2) | Mismatch repair | 1.37E-06 |
| Pre B->immature B(B2) | Hematopoietic cell lineage | 1.37E-05 |
| Pre B->immature B(B2) | B Lymphocyte Cell Surface Molecules | 9.38E-05 |
| Pre B->immature B(B2) | Base excision repair | 2.70E-04 |
| Immature B->B(B3) | Pathways in cancer | 6.13E-09 |
| Immature B->B(B3) | Apoptosis | 2.94E-07 |
| Immature B->B(B3) | Cell cycle | 4.45E-07 |
| Immature B->B(B3) | B cell receptor signaling pathway | 3.62E-06 |
| Immature B->B(B3) | Fc epsilon RI signaling pathway | 6.23E-06 |
| Immature B->B(B3) | Toll-like receptor signaling pathway | 1.32E-05 |
| DN-.>DP(T1) | Hematopoietic cell lineage | 1.32E-06 |
| DN-.>DP(T1) | Primary immunodeficiency | 2.10E-04 |
| DN-.>DP(T1) | Cell adhesion molecules (CAMs) | 4.66E-04 |
| DN-.>DP(T1) | Lck and Fyn tyrosine kinases in initiation of TCR Activation | 0.003974183 |
| DN-.>DP(T1) | Antigen processing and presentation | 0.00481351 |
| DN-.>DP(T1) | T cell receptor signaling pathway | 0.005801918 |
| DP->CD4+(T2) | Hematopoietic cell lineage | 2.67E-05 |
| DP->CD4+(T2) | Cell cycle | 6.20E-04 |
| DP->CD4+(T2) | Oocyte meiosis | 0.003979894 |
| DP->CD8+(T3) | Hematopoietic cell lineage | 3.92E-05 |
| DP->CD8+(T3) | Cell cycle | 5.41E-04 |
| DP->CD8+(T3) | p53 signaling pathway | 0.00346149 |
| DP->CD8+(T3) | Oocyte meiosis | 0.00609765 |
| DP->CD8+(T3) | Progesterone-mediated oocyte maturation | 0.009368626 |

**Supplementary Table S2: The top 10 TFs/miRNAs based on the number of B cell related papers from literature mining.**

| **B-TF** | **Number of papers** | **B-miRNA** | **Number of papers** |
| --- | --- | --- | --- |
| MYC | 1785 | miR-155-5p | 96 |
| BCL6 | 730 | miR-17-5p | 57 |
| PAX5 | 492 | miR-15a-5p | 30 |
| TCF3/E2A | 320 | miR-150-5p | 26 |
| SPIB/PU.1 | 245 | miR-16-5p | 26 |
| IRF4 | 211 | miR-34a-5p | 20 |
| BLNK | 143 | miR-20a-5p | 12 |
| RUNX1 | 124 | miR-142-3p | 9 |
| MYB | 119 | miR-19a-3p | 9 |
| EBF1 | 115 | miR-106a-5p | 8 |

Red represents hub TFs/miRNAs.

**Supplementary Table S3: The top 10 TFs/miRNAs based on the number of T cell related** papers from literature mining.

| **T-TF** | **Number of papers** | **T-miRNA** | **Number of papers** |
| --- | --- | --- | --- |
| FOXP3 | 4828 | miR-155-5p | 101 |
| REL | 333 | miR-17-5p | 43 |
| GATA3 | 273 | miR-21-5p | 41 |
| MYB | 203 | miR-146a-5p | 35 |
| TCF3/E2A | 187 | miR-223-3p | 30 |
| RUNX1 | 114 | miR-181a-5p | 26 |
| SPI1/PU.1 | 114 | miR-150-5p | 23 |
| IRF4 | 99 | miR-142-3p | 17 |
| BCL11B | 83 | miR-125b-5p | 10 |
| LEF1 | 75 | miR-16-5p | 10 |

Red represents hub TFs/miRNAs.
